# Involvement of Dentists in Preventing Early Childhood Caries in Germany

**DOI:** 10.3390/medicina61111947

**Published:** 2025-10-30

**Authors:** Abdullah Takriti, Antje Geiken, Christian Graetz, Christof E. Doerfer, Mhd Said Mourad, Christian H. Splieth

**Affiliations:** 1 Department Pediatric Dentistry, University Greifswald, 17489 Greifswald, Germany; abdullah.takritti@gmail.com (A.T.); splieth@uni-greifswald.de (C.H.S.); 2 Clinic for Conservative Dentistry and Periodontology, University Schleswig-Holstein, 24105 Kiel, Germany; geiken@konspar.uni-kiel.de (A.G.); graetz@konspar.uni-kiel.de (C.G.); christof.doerfer@uksh.de (C.E.D.); 3 Department of Orthodontics, University Greifswald, 17489 Greifswald, Germany

**Keywords:** early childhood caries, preventive dentistry, fluoride toothpastes, oral hygiene, children

## Abstract

*Background and Objectives:* Early Childhood Caries affect children’s quality of life and overall health. This study aimed to assess the involvement of dentists in implementing early preventive measures, including fluoride use, for children aged 6–33 months. *Materials and Methods:* a multiple-choice questionnaire was distributed in six German states, consisting of two sections: Section I covered participant demographics and Section II included items on dental preventive measures for children. The questionnaire was adapted from a validated German-language source, reviewed by five experts at Kiel University, and tested in a focus group of 30 dentists. Descriptive statistics (mean ± SD or median [IQR]) and Mann–Whitney U tests were used to assess pediatric dentists (PD) and general dentists (GP) involvement in early dental preventive measures. *Results:* A total of 511 eligible questionnaires were returned (mean age 47 ± 11 year, 63.8% females, 36.7% PD). Both GP and PD routinely recommended a diagnostic dental visit (1 = never, 5 = always), with PD reporting higher frequency (GP: 4 [3–5], PD: 4.5 [4–5]; *p* = 0.001). Parental training in oral hygiene was performed significantly more often by PD (*p* < 0.01). PD also recommended tooth brushing with fluoridated toothpaste after the eruption of the first tooth more frequently than GP (GP: 5 [3–5], PD: 5 [4–5]; *p* = 0.06). Surprisingly, fluoride-free toothpaste was still recommended by a relevant number of respondents in both groups. *Conclusions:* PDs showed greater involvement in early caries prevention than GP. While most recommended fluoridated toothpaste, many still advised fluoride-free options, highlighting gaps in guideline adherence.

## 1. Introduction

Early Childhood Caries (ECC) is considered one of the most significant untreated health problems in preschool children in both industrial and developing countries, which affects the quality of life and development of the children, and causes pain as well as possible psychological problems [1,2].

The prevalence of ECC in Germany differs only minimally between the different federal states and represents a relevant problem in 3-year-olds, with a prevalence of 10–17% [3]. Because of this high number of affected primary teeth, the severity, the young age of the children, and consequently their low ability to cooperate, Early Childhood Caries represents a major dental challenge in young children. Especially in severe forms, often only the extraction of the affected teeth under dental general anesthesia is possible [3].

The rising costs and risks of the general anesthesia treatment in children with ECC highlight the importance of early dental prevention measures targeting children from the eruption of the first primary teeth [4]. The interval of the dental visits should take into consideration the child’s individual needs, caries risk, and caries activity status, considering the patient’s clinical history and the radiographic and clinical findings [5].

In addition to inadequate oral hygiene, ECC is strongly associated with improper feeding practices, such as prolonged bottle-feeding, frequent intake of sugary drinks, and the use of pacifiers dipped in sweet substances commonly referred to as nursing bottle caries or baby bottle tooth decay [6,7,8,9]. These modifiable behavioral factors increase caries risk significantly and are preventable through early parental counseling.

Many studies showed that fluoridated toothpaste is an effective measure, given its low cost and caries-preventive effect [10,11,12,13,14]. Therefore, as recommended by the European Organization for Caries Research (ORCA), the European Federation of Conservative Dentistry (EFCD), the European Academy of Paediatric Dentistry, and the German Society for Preventive Dentistry (DGPZM), parents or caregivers should brush their children’s teeth daily with an age-appropriate fluoridated toothpaste as soon as the first primary tooth erupts [15,16,17].

Both pediatric dental specialists (PD) and general dentists (GD) play an important role in preventing Early Childhood Caries (ECC) by conducting regular dental examinations, implementing caries prevention strategies, and educating parents about appropriate dietary habits for young children.

To our knowledge, only limited international data and none from Germany have explored dentists’ involvement in the prevention of Early Childhood Caries (ECC). Therefore, the aim of this study was to assess the involvement of dentists in Northern German states in implementing various preventive measures related to ECC.

## 2. Materials and Methods

### 2.1. Study Design and Sample

A structured, multiple-choice questionnaire was developed in German, drawing on elements from previously used instruments in the field [18,19]. The questionnaire underwent expert review by five specialists in pediatric dentistry and web-based survey methodology at Kiel University to ensure content relevance, clarity, and face validity. It was subsequently implemented as an online survey using a standardized platform (Unipark, QuestBack GmbH, Cologne, Germany). To assess comprehensibility and feasibility, the questionnaire was pilot tested in a focus group consisting of 30 practicing dentists.

This questionnaire was divided into two sections: Section I dealt with general information about the participants (age, gender, field of strength, etc.), while Section II contained multiple items related to dental preventive measures and recommendations provided in the dental practice for young children between 6 and 33 months. This part consisted of statements to be answered with a 5-point Likert scale (1 = never to 5 = always). Ethical approval was obtained from the ethical committee of Christian–Albrechts-University in Kiel (No: D452/18).

After contacting the dental associations of the German states, the following states were interested in participating in our study (Hamburg, Lower Saxony, Mecklenburg-Western Pomerania, Schleswig-Holstein, Berlin, Germany), and they approved to publish an invitation for dentists to participate in the study in their online Newsletter. The participants were then able to fill in an online version of the questionnaire, which was designed by the web-based questionnaire tool (Unipark, QuestBack GmbH, Cologne, Germany). The questionnaire was activated online from 20 March until 8 August 2019. In addition, it was possible to distribute the questionnaire during the 26th Schleswig-Holstein dental conference, where participants from the above-mentioned states attended.

Participation in the study was entirely voluntary. By completing and returning the anonymous questionnaire, participants provided implied informed consent. They were informed that participation was optional and that they could choose not to complete or return the questionnaire without any consequences.

### 2.2. Inclusion Criteria

All dentists who owned or worked in a dental practice in the participating states were included. Partially answered questionnaires were accepted and included in the statistical analysis, if the answers were logical (in which all personal data were answered and no answer contradicts the other answers), and the age, sex, as well as the federal state where the dentists worked were reported. Dentists who worked in universities, the Ministry of Health, or who had retired were excluded.

### 2.3. Statistical Analysis

Descriptive statistics were presented as mean and standard deviation for normally distributed variables, and as median and interquartile range (IQR; 25th–75th percentile) for non-normally distributed variables. Normality was assessed using the Kolmogorov–Smirnov test. Group comparisons were performed using the Mann–Whitney U test for non-parametric data. All statistical analyses were conducted using IBM SPSS Statistics for Windows, Version 26.0 (IBM Corp., Armonk, NY, USA).

## 3. Results

During the 26th Schleswig-Holstein Dental Conference, a total of 711 questionnaires were distributed. Of these, 477 were completed and returned either in person at the conference or via post, email, or fax, resulting in a response rate of 67.1%. After applying the inclusion criteria, 349 of the returned questionnaires (73.2%) were included in the final analysis (349/477).

In addition to the 477 completed and returned paper questionnaires, the online version of the questionnaire was completed by another 162 dentists. This resulted in a total of 511 questionnaires (paper and online combined) included in the final analysis.

### 3.1. Characteristics of the Study Sample

The mean age of the participants was 47 years (Table 1), with 63.8% being female. 36.7% (n = 188) of the participants were dentists with a specialized paediatric background or training. While 69% of the participants spend only 0–5% of their daily practicing time treating children under 33 months of age (Table 1).

### 3.2. Difficulties Faced During an Early Preventive Dental Visit

The lack of cooperation during the dental examination was found to be the main difficulty faced by more than half of the participating dentists (58.7%, Table 2). Only 4 (0.7%) of the participants stated that a dental examination at this age is not necessary, as the children are still too young. These were all dentists without a background in paediatric dentistry (Table 2).

### 3.3. Implemented Dental Activities and Measures in the Dental Office

Participants were asked about dental activities and preventive measures implemented in their practices for children under 33 months of age. This section included five statements addressing this topic (Table 3). Pediatric dental specialists (PD) were significantly more likely than general dentists (GD) to perform early dental examinations (PD: 4.5 [3–5], GD: 4.0 [3–5]; *p* = 0.001, Mann–Whitney U test).

PD also provided significantly more parental education and discussions on the importance of oral hygiene procedures compared to GD (PD: 5 [4–5], GD: 5 [4–5]; *p* = 0.000, Mann–Whitney U test), suggesting that PD were more actively involved in implementing early preventive measures, including nutritional counseling, parental guidance, and oral hygiene education.

### 3.4. Recommendations Regarding the Use of Fluoridated Toothpaste

In the second section of the questionnaire, participants were asked about recommendations regarding fluoridated toothpaste. The questions included different recommendations concerning the age of the child and the kind of recommended toothpaste. PD appeared to recommend fluoridated toothpaste with the eruption of the first primary tooth more often than GD, with no significant difference found between the groups (PD: 5 [4–5], GD: 5 [3–5]; *p* = 0.06, Mann–Whitney U test). Both PD and GD recommended the use of fluoride-free toothpaste by a relevant number of participants (median score: 1 [1–2]; Table 4).

## 4. Discussion

Early Childhood Caries, and according to prevention methods, has been a subject for discussion in recent years in Germany. In spite of the caries decline in the permanent dentition, caries in the primary dentition is still a major challenge [3]. This is most likely associated with the fact that preventive activity in the German National Health System concentrated on the permanent dentition, that children do not visit the dentist at an early age, and many parents/caregivers are not aware of the importance of these visits and the role of early prevention in keeping and providing a healthy dentition for their children.

In Germany, visiting the paediatrician from an early age, directly after the birth, is considered obligatory and covered by the insurance [20]; while visiting the dentist is optional, and preventive activities in children below 30 months of age were only introduced in July 2019 [21].

The high prevalence of ECC highlights the importance of early dental prevention and examination measures in children starting from the eruption of the first primary teeth. This was considered a challenging task for dentists, due to the lack of cooperation in small children, which is in line with other studies reported in the literature [22].

Thus, dentist training and education play a significant role in performing prevention in small children. The lack of experience may create a barrier and prevent dentists from treating and providing proper dental health care for young children [23]. Unfortunately, German universities do not have a unified national undergraduate curriculum, and many universities do not offer any clinical training in the field of paediatric dentistry at all [24].

This study showed that recommendations given by many dentists can be considered good, but it is not satisfying that only 44.2% performed early preventive dental visits for children between 6 and 33 months as a routine activity in their dental office.

This contradicts the recommendations and the guidelines of many international dental associations such as the one from the European Organization for Caries Research (ORCA) and European Federation of Conservative Dentistry (EFCD), the European Academy of Paediatric Dentistry (EAPD), and the American Academy of Pediatric Dentistry (AAPD) which highly recommend visiting the dentist within the first six months and informing the parent about etiology and prevention of dental caries [16,25,26].

Our study showed that only 15.3% of dentists were always training parents to apply oral hygiene procedures to their children. This low number could be an indicator of a serious problem in applying a correct dental preventive program, regarding the fact that parents are the main caregivers for their children at this age. This result corresponds to the result of another study, which concluded that further oral health education is needed [27]. Dentists with specialized training in paediatric dentistry seemed to perform early preventive dental visits in children from six months on more often than GD. This difference was not limited to the early dental examination itself, but also seen in all preventive measures in this age group, which corresponds to the results of another study, which also concluded that both GD and PD are applying the early diagnostic and preventive examination, in which the PD are more involved in the preventive and fluoride measures [28].

While our study is the first to assess the involvement of dentists in ECC prevention across multiple German states, similar efforts have been undertaken internationally. For example, a French study found that although pediatricians and general practitioners were interested in ECC detection, many lacked full knowledge of dietary risk factors and the optimal referral age [29]. This aligns with our findings that emphasize the need for better preventive training among healthcare providers. Studies from India and other countries have also shown delayed first dental visits, often after caries onset, and highlight gaps in parental awareness, mirroring our observation that only 44.2% of dentists reported routinely conducting early preventive visits [30,31,32]. Moreover, challenges in oral hygiene education and inconsistent fluoride recommendations were similarly noted in those settings, reinforcing the global need for harmonized ECC prevention strategies. These cross-national findings support the relevance and urgency of improving early prevention efforts and provider training, particularly among general dentists.

Another important cornerstone in the prevention of ECC is the use of fluoridated toothpaste. Many studies and a recent Cochrane review underlined the effectiveness of fluoridated toothpaste [10]. In our study, the recommendations given by participants could be interpreted as adequate, as more than half of them, 55.7% (n = 285), proposed fluoridated toothpaste according to the latest fluoride recommendations of the American and European Academies of Paediatric Dentistry and the German Society of Preventive Dentistry [15,17,33]. However, there is still a need to improve the use of fluoridated toothpaste for this age group, as some dentists still recommend fluoride-free toothpaste. The reason behind this was difficult to identify, as our questionnaire was not designed to investigate the underlying reasoning of the dentists. Another possible reason may be that most of the dentists still consider the old fluoride recommendations of paediatricion with Fluoride tablets, and consider it as enough, and may substitute the prescription of fluoridated toothpaste [34].

While the use of fluoridated toothpaste remains the gold standard for caries prevention in children, concerns have been raised about the systemic effects of fluoride, particularly when ingested in large quantities [10,35]. Evidence shows that water fluoridation effectively reduces caries prevalence, but may be associated with an increased risk of dental fluorosis, most of which is mild and not of aesthetic concern [35]. Current evidence does not support a clear link between optimally fluoridated water and increased risks of cancer, bone fracture, or other systemic harms [35,36]. In contrast, topical fluoride agents, such as toothpaste and varnish, have shown strong caries-preventive effects with minimal systemic absorption [10,36]. Although some studies suggest a possible association between topical fluoride use and “any fluorosis,” the risk of fluorosis of aesthetic concern remains very low when used appropriately. Recent research into fluoride alternatives, such as hydroxyapatite (HAP)-based toothpaste, has shown promising caries-preventive potential, though more high-quality comparative studies are needed [37]. Nonetheless, current guidelines from leading dental organizations continue to recommend fluoridated toothpaste, used in age-appropriate amounts, as the safest and most effective approach for ECC prevention in young children [15,38].

The results of our study support the importance of the recent introduction of free-of-charge preventive dental visits for 6–33-month-old children in the German National Health system in July 2019, which includes the assessment of plaque and gingivitis, other ECC risk factors, diet, and fluoride counseling, as well as brushing training [39].

This may have a positive effect on the implementation of ECC prevention, and it could encourage general dentists to intensify their efforts in this field. It seems that such programs can improve medical care regardless of being performed by general or paediatric dentists [40].

Although this questionnaire was primarily distributed and completed by dentists from six German federal states, the results may still be generalizable to other regions, as national regulations and insurance policies related to pediatric dental care are consistent across Germany.

As no accurate or up-to-date data were available regarding the total number of dentists actively treating children in the participating federal states, a formal sample size calculation could not be performed. Therefore, the study followed an exploratory design using convenience sampling. Our aim was to include as many eligible dentists as possible by distributing the questionnaire through state dental associations and at a major regional dental congress. As with all questionnaire-based studies, there is a potential for selection bias. However, the relatively large sample size and the alignment of key findings in the existing literature enhance the plausibility and relevance of our results.

A further limitation is that the study did not include a structured multi-criteria decision-making (MCDM) approach to assess the relative importance of different preventive strategies. Future research could benefit from applying MCDM methods to rank ECC prevention measures based on effectiveness, feasibility, practitioner preferences, and alignment with clinical guidelines.

## 5. Conclusions

Both general dentists and pediatric dental specialists demonstrated engagement in Early Childhood Caries prevention, with pediatric dentists showing significantly higher involvement in areas such as diagnostic visits and parental oral hygiene education. However, recommendations regarding fluoride use and timing were not always in line with current national or international guidelines. While most dentists recommended fluoridated toothpaste after the eruption of the first tooth, a considerable number still advised fluoride-free alternatives, highlighting the need for improved adherence to evidence-based recommendations. Nonetheless, the findings indicate a strong foundation for the recently introduced, cost-free ECC prevention measures, especially among dentists with pediatric training.

## Figures and Tables

**Table 1:** Characteristics of the study sample of PD and GP dentists in absolute values and in percentages (%).

**Characteristics of the Study Sample**						
**gender, n (%)**	**Female**		**Male**		**Total**	
	326 (63.8)		184 (36.0)		510 (100)	
**age, mean (SD)**	44.4 (10.2)		50.9 (11.4)		47 (11.0)	
**Comparison of Demographic Characteristics**						
**Variable**	**PD (n = 188)**		**GP (n = 323)**		***p*** **-Value**	
**Age median (Q1–Q3)**	48 (38–56)		47 (38–56)		0.272 *	
**Female, n (%)**	139 (73.9%)		187 (57.9%)		0.001 **	
**Consumed Treating Children Under 33 Months**						
**Daily Time in %**	**0–5%**	**5–10%**	**10–15%**	**15–20%**	**>20%**	n.a. ***
**n (%)**	353 (69.0)	98 (19.1)	25 (4.8)	14 (2.7)	18 (3.5)	3 (0.5)
**Specialty and Field of Strength**						
**PD, n (%)**		188 (36.7)				
**GD, n (%)**		323 (63.3)				

* Mann–Whitney U test; ** Chi-squared test; *** No answer.

**Table 2:** Difficulties faced during an early preventive dental visit of 6–33-month-old children in absolute values and in percentages (%).

Difficulty Faced	PD n (%)	GP n (%)
**low cooperation during the dental examination**	97 (51.6)	203 (63.4)
**children are bothered by having dental instruments in their mouths**	65 (34.6)	162 (50.6)
**little cooperation/interest of parents**	54 (28.7)	66 (20.6)
**parents do not keep appointments**	48 (25.5)	61 (19.0)
**insufficient dental experience in dealing with young children**	14 (7.4)	36 (11.2)
**it is not necessary because the children are still too young**	0 (0)	4 (1.2)
**I do not see any difficulty**	63 (33.5)	74 (23.1)

**Table 3:** Implemented dental activities and measures in the dental office in absolute values and in percentages (%).

Activities and Measurements Performed in Young Children	Total Answered	PD			GP			*p*-Value *
	n (%)	n	Median (Q1–Q3)	Mean (SD)	n	Median (Q1–Q3)	Mean (SD)	
**early dental examination**	503 (98.4)	184	4.5 (3–5)	4.0 (±1.1)	319	4 (3–5)	3.6 (±1.2)	0.001 **
**educating parents about caries prevention**	505 (98.8)	186	5 (4–5)	4.5 (±0.7)	319	5 (4–5)	4.3 (±0.8)	0.004 **
**educating parents regarding oral hygiene practices**	505 (98.8)	184	5 (4–5)	4.6 (±0.6)	321	5 (4–5)	4.3 (±0.8)	0.000 **
**training of parents for oral hygiene measures on their own child**	501 (98.0)	184	5 (3–5)	3.4 (±1.2)	317	3 (2–3.5)	2.6 (±1.2)	0.000 **
**nutritional advice related to caries prevention**	503 (98.4)	185	5 (4–5)	4.2 (±0.8)	318	4 (3–5)	3.9 (±1.0)	0.000 **

* Mann–Whitney U test.; ** statistically significant.

**Table 4:** Recommendations regarding the use of fluoridated toothpaste by paediatric dentists and general dentists in paediatric dentistry in absolute values and in percentages (%).

Recommendation	Total Answered	PD			GP			*p*-Value *
	n (%)	n	Median (Q1–Q3)	Mean (SD)	n	Median (Q1–Q3)	Mean (SD)	
**children fluoridated toothpaste with the eruption of the first primary tooth (6th–8th months)**	499 (97.6)	184	5 (4–5)	4.1 (±1.2)	315	5 (3–5)	3.9 (±1.4)	0.06
**children fluoridated toothpaste after the complete eruption of all primary teeth**	465 (90.9)	167	1 (1–2)	1.7 (±1.3)	298	1 (1–2)	1.6 (±1.2)	0.22
**children fluoridated toothpaste, only when the child can already spit out well**	467 (91.3)	164	1 (1–3)	2.1 (±1.5)	303	1 (1–3)	2.0 (±1.5)	0.37
**junior or adult fluoridated toothpaste for children under 6 years of age**	473 (92.5)	166	1 (1–3)	2.1 (±1.4)	307	1 (1–2)	1.8 (±1.2)	0.008 **
**fluoride-free toothpaste**	473 (92.5)	167	1 (1–2)	1.7 (±1.2)	306	1 (1–2)	1.7 (±1.3)	0.55

* Mann–Whitney U test; ** statistically significant.

## Data Availability

The data sets presented in this article are not publicly available due to data protection regulations. Requests for access should be directed to the corresponding author.
